# Challenges to be overcome using population-based sampling methods to recruit veterans for a study of post-traumatic stress disorder and traumatic brain injury

**DOI:** 10.1186/1471-2288-14-48

**Published:** 2014-04-08

**Authors:** Peter J Bayley, Jennifer Y Kong, Drew A Helmer, Aaron Schneiderman, Lauren A Roselli, Stephanie M Rosse, Jordan A Jackson, Janet Baldwin, Linda Isaac, Michael Nolasco, Marc R Blackman, Matthew J Reinhard, John Wesson Ashford, Julie C Chapman

**Affiliations:** 1 War Related Illness and Injury Study Center (WRIISC), Veteran Affairs Palo Alto Health Care System, 3801 Miranda Avenue (151Y), Palo Alto, CA 94304-1290, USA; 2 Department of Psychiatry and Behavioral Sciences, Stanford University School of Medicine, Stanford, CA, USA; 3 WRIISC, Veteran Affairs New Jersey Health Care System, East Orange, NJ; University of Medicine & Dentistry, Rutgers University, New Jersey Medical School, Newark, NJ, USA; 4 Department of Veterans Affairs, Epidemiology Program, Office of Public Health, Washington, DC, USA; 5 WRIISC, Veteran Affairs Medical Center, Washington, DC, USA; 6 Research Service, Veterans Affairs Medical Center, Washington, DC, USA; 7 Departments of Medicine and Rehabilitation Medicine, Georgetown University School of Medicine, Washington, DC, USA; 8 Departments of Medicine, Biochemistry and Molecular Medicine, George Washington University School of Medicine, Washington, DC, USA; 9 Department of Psychiatry, Georgetown University School of Medicine, Washington, DC, USA; 10 Department of Neurology, Georgetown University School of Medicine, Washington, DC, USA

**Keywords:** MIND study, PTSD, Recruitment methods, Recruitment yields, TBI, Veteran

## Abstract

**Background:**

Many investigators are interested in recruiting veterans from recent conflicts in Afghanistan and Iraq with Traumatic Brain Injury (TBI) and/or Post Traumatic Stress Disorder (PTSD). Researchers pursuing such studies may experience problems in recruiting sufficient numbers unless effective strategies are used. Currently, there is very little information on recruitment strategies for individuals with TBI and/or PTSD. It is known that groups of patients with medical conditions may be less likely to volunteer for clinical research. This study investigated the feasibility of recruiting veterans returning from recent military conflicts— Operation Enduring Freedom (OEF) and Operation Iraqi Freedom (OIF) - using a population-based sampling method.

**Methods:**

Individuals were sampled from a previous epidemiological study. Three study sites focused on recruiting survey respondents (n = 445) who lived within a 60 mile radius of one of the sites.

**Results:**

Overall, the successful recruitment of veterans using a population-based sampling method was dependent on the ability to contact potential participants following mass mailing. Study enrollment of participants with probable TBI and/or PTSD had a recruitment yield (enrolled/total identified) of 5.4%. We were able to contact 146 individuals, representing a contact rate of 33%. Sixty-six of the individuals contacted were screened. The major reasons for not screening included a stated lack of interest in the study (n = 37), a failure to answer screening calls after initial contact (n = 30), and an unwillingness or inability to travel to a study site (n = 10). Based on the phone screening, 36 veterans were eligible for the study. Twenty-four veterans were enrolled, (recruitment yield = 5.4%) and twelve were not enrolled for a variety of reasons.

**Conclusions:**

Our experience with a population-based sampling method for recruitment of recent combat veterans illustrates the challenges encountered, particularly contacting and screening potential participants. The screening and enrollment data will help guide recruitment for future studies using population-based methods.

## Background

Achieving patient enrollment targets in clinical research is one of the main challenges facing researchers today. The magnitude of the challenge is not well documented but according to one review that examined 41 clinical trials in the US, 34% of the trials recruited less than 75% of their planned sample [1]. Under-recruitment reduces the statistical power of the study and often leads to inconclusive results [2-4]. Remediation of under-recruitment to avoid study closure often leads to costly changes including a re-evaluation of the sample size required, extension of the patient intake period, modification of the inclusion/exclusion criteria, and addition of new study sites [5].

Clinical studies utilizing population-based sampling methods are particularly challenging in this regard as they rely upon the cooperation of a predetermined sample, members of which have no knowledge of the study prior to the first contact. The restricted pool of potential participants places additional importance on engaging each participant as a representative of the population relative to studies using convenience-based sampling methods. For example, a study of Gulf War Veterans [6] used a population-based sampling method to recruit a group of veterans who had been deployed to the Persian Gulf between 1990 and 1991. Twenty-one VA sites participated and reported that only 13.7% of potential participants were successfully contacted. However, population-based sampling methods offer the potential for high external validity such that the results can be applied directly to the defined population [7]. Use of techniques such as random selection of population members and invitation of greater proportions of high-interest subgroups, for example, increase the potential representativeness of such a sample. For these reasons, studies which estimate prevalence rates in the reference population and identify trends or risk factors frequently utilize this method. Population-based sampling methods can be highly effective for studies in which the sampling frame is clearly defined and all required data is recorded in a database or a medical record system. However, when human participation is required, the effectiveness of population sampling methods depends upon the willingness of the individuals to become involved in the study. These individuals may share some similarities which may introduce bias into the sample such as being the most severely impacted by the condition being investigated or having the least access to medical care. Other factors impacting the decision to participate include competing interests and time constraints, as well as concerns about risks and safety [8,9], fear of additional financial costs [10,11], concerns about randomization [12], loss of confidentiality [13], religious and cultural reservations [14,15], and stigma associated with disease [16,17]. Thus, although the initial random selection in a population-based sample may offer some preliminary protection from bias, responses may still be systematically biased, posing a threat to external validity with weaknesses that may rival convenience based sampling strategies.

The current pilot study entitled “Markers for the Identification, Norming, and Differentiation of TBI and PTSD” (MIND study) was designed as a clinical sub-study of a longitudinal study “National Health Study for a New Generation of US Veterans” (“New Generation Study”). The New Generation Study [18] was one of the first epidemiologic studies to examine the health outcomes of veterans returning from recent conflicts in Afghanistan and Iraq —Operation Enduring Freedom (OEF) and Operation Iraqi Freedom (OIF). The study began in 2009 and involved web-based, postal, or computer assisted telephone interviews to survey a stratified random sample of 60,000 veterans with documented military service between October 2001 and June 2008. The population sample included 30,000 veterans who had deployed and 30,000 non-deployed veterans who had served during the same era. Recruitment was achieved via a 72-item survey of veterans who had left combat theaters (reserve and National Guard) or separated from active duty by June 2008. The sample was stratified by deployment status, gender, unit component (active duty, reserve, National Guard), and branch of service (Army, Air Force, Navy, Marines). Women veterans were oversampled to comprise 20% of potential participants, reflecting an increased awareness of women’s unique health concerns [19]. The primary objective of the New Generation Study was prevalence estimation of health concerns specific to this cohort including traumatic brain injury (TBI) and posttraumatic stress disorder (PTSD). The findings revealed that those who deployed to Afghanistan, Iraq, and other countries had higher estimated prevalence of TBI, PTSD, and co-occurring TBI/PTSD than those who were not deployed (A. Schneiderman, personal communication). These results and other studies [20-22], reflect the now widely held view that TBI and PTSD are important consequences of the OEF/OIF/Operation New Dawn conflicts.

The MIND study was designed to be conducted at the War Related Illness and Injury Study Center (WRIISC). The main aim of the MIND study was to determine the prevalence of TBI and PTSD, to identify sensitive and specific measures for them, and to develop prediction models. The WRIISC, located in three sites (Washington, DC, Palo Alto, CA and East Orange, NJ) focuses on post-deployment health concerns of veterans for the Veterans Health Administration (VHA). The WRIISC provides in depth clinical assessment and diagnostics for unexplained symptoms, chronic multi-symptom illness, or treatment-resistant illness or injury that is putatively related to or exacerbated by their deployment.

The current study was conceived as a MIND pilot with a view to conducting a larger study involving 800 veterans. Participation in the MIND pilot study involved travelling to one of the three WRIISC study sites for a full day of assessment that included cognitive and sensorimotor testing, brain imaging and psychiatric evaluation. Since human studies using population-based sampling methods frequently have relatively low recruitment rates, one of the primary objectives of the current study was to demonstrate that adequate numbers of individuals from the New Generation Study could be contacted and successfully recruited. For the MIND pilot study, potential participants were recruited via the US Postal Service with subsequent direct telephone contact; the effectiveness of these methods was evaluated.

A secondary objective of the current study was to examine the association between recruitment, screening, and enrollment and the individuals’ characteristics from the New Generation Study. Some individuals may be less likely to volunteer for clinical research or participate in the health care system more generally either as a consequence of their medical symptoms or as a result of the stigma associated with illness (for discussions, see [16,17]). Accordingly, we also examined recruitment rates from four self-reported diagnostic categories from the New Generation Study (probable TBI, probable PTSD, probable TBI and PTSD, and probable neither condition).

## Methods

### Participants

An initial sample of 651 veterans (529 males, 122 females) was identified in 2012 for potential participation in the MIND pilot study (mean age = 39.1 yr, range = 26.7 to 52.8 yr). The criteria for inclusion in the sample to be contacted were: i) participation in the New Generation Study, ii) deployed to OEF/OIF conflicts for ≥ 30 days, and iii) likelihood of residence location within a 60-mile radius of one of the three WRIISC centers. Using the addressees at which New Generation participants were successfully enrolled, zip code matching identified potential participants based on probable locations within 60 miles of the three WRIISC sites. To assess the quality of addresses on record and secure likely telephone numbers for possible follow-up, the participant list was matched against a LexisNexis Corporation database to identify up to eight additional addresses and ten telephone numbers per individual, listed in order of likelihood of being a current match.

### Procedure

The screening protocol was approved by the Institutional Review Board (IRB) and Research and Development Committees (RDC) associated with each of the three WRIISC sites (Veterans Affairs Palo Alto Healthcare System, CA, the Veterans Affairs New Jersey Healthcare System in East Orange, NJ, and the Washington DC Veterans Affairs Medical Center). In order to increase the likelihood of contacting individuals who lived locally, a further criterion was applied to the sample of 651 potential participants, namely, that their “best address” identified by the computer assisted address search was within 60 miles of one of the three study sites. This restriction yielded 445 individuals (DC 189, NJ 189, CA 67). During the course of screening, it was noted that the usefulness of the addresses and telephone numbers returned by LexisNexis diminished rapidly after the third listed contact and for this reason we did not use the information beyond this point.

An “Advance Letter” signed by the principal investigator was sent to each of the 445 individuals at their “best address”. The letter briefly described the study and invited them to contact the local study coordinator to request to be screened or to opt out of any further contact. The letter stated that compensation would be given for participation and mileage. Telephone screening was delayed for two weeks after the Advance Letters were mailed in order to provide candidates sufficient time to opt out of the study before being contacted. Of note, all of these individuals had been successfully contacted in the New Generation Study by mail between 2009 and 2011 and had returned completed surveys.

### Screening procedure

The purpose of screening was to establish eligibility for enrollment (see Additional file 1 for criteria) and to collect information related to demographics, general health, TBI, and symptoms of PTSD. Major inclusion criteria were age 18 to 50 years and deployment for ≥30 days in the OEF/OIF conflict. As the objectives of the MIND study were to determine the prevalence of PTSD, and to identify sensitive and specific measures, we recruited individuals that reached full (currently utilized) diagnostic criteria, rather than individuals with sub-threshold symptoms. Screening was conducted over a total of 23 weeks. Telephone screening was conducted by experienced research staff who were trained and supervised on the MIND protocol. The semi-structured screening interview contained 117 items. Due to the sensitive nature of the interview (e.g., items about suicidal ideation), a safety plan and a response algorithm were developed in the event that screening participants expressed emotional distress, any suggestion of suicidal or homicidal intent, or conditions requiring immediate medical attention. Senior study and clinical staff were always available during screening.

Each study site focused on local recruitment. The frequency and timing of screening calls was designed to maximize the possibility of successfully contacting a potential participant. Each individual was called once per week with the day and time of day recorded. If the call was not answered, a discrete voicemail message was left stating who the call was from and the contact number of the study coordinator, omitting any details that would impact the individual’s privacy.

Once initial contact had been established, the call began with a short description of the MIND study. The individual was asked if they were interested in participating and if they would like to continue onto the phone screening. If they were interested in the study, they were told that the compensation rate was $200 plus travel expenses, and overnight accommodation in a hotel if their home address was >50 miles from the study center. Some individuals were screened on the same call, while others were interested in being screened but made an appointment for a later time. If we were unable to get in contact with these individuals again after the initial call, they were categorized as “initial contact only” (Table 1).

**Table 1:** The frequency of individuals who were successfully contacted as a function of group membership in the New Generation Study

		**New generation group membership**				**Total**
		**TBI**	**PTSD**	**TBI and PTSD**	**Neither TBI nor PTSD**	
Contacted and screened (n)		4	8	15	39	66
Contacted but not screened (n)		15	8	9	47	79*
χ^2^ (screened vs. not screened)		7.88**				
df		3				
Reason not screened	Not interested	8	1	5	23	37
	Initial contact only	6	5	3	16	30
	Too Far	1	2	0	7	10
	Pregnancy	0	0	0	1	1
	Deployment	0	0	1	0	1

*One of the 80 individuals contacted but not screened did not have a New Generation study diagnosis. **Fewer individuals with a diagnosis of TBI were screened than would be expected by chance alone (p<.05).

At the beginning of the screening call, participant identity was verified by asking their date of birth and the last 4 digits of their social security number and comparing these values with those on record. All screening questions were then asked unless the participant requested to stop. Screening data were reviewed following the call and participants were again contacted by telephone as soon as possible –usually within a day-- regarding their eligibility and continued interest in the study. If the study criteria were met and the individual chose to participate, he/she was invited to the study site nearest his/her home for an in-person assessment. If the individual did not wish to participate in the study or the study criteria were not met, he/she was thanked for answering the screening questions and the call was ended.

### Statistical analysis

All analyses presented are exploratory and were performed using SPSS (v 18.0.2) with a significance value of 0.05 (two-tailed). Chi-square tests were computed to compare frequencies of patients across different probable diagnostic groups determined by their answers to the screening items of the New Generation Study postal survey (i.e., probable TBI, probable PTSD, probable PTSD and TBI, probable neither). If the expected count in any cell was <5, Fisher’s exact test was used instead. Group differences were identified by examination of the standardized residuals for which a value > ±1.96 represents a significant difference between groups.

Comparisons between groups involving continuous variables (e.g., age, distance from study site, driving distance from study site) were made using unpaired t-tests. The distance between the participant’s home address and the study site was generated for individuals whose address was confirmed using a web-based distance calculator (http://www.zip-codes.com).

## Results

**Figure 1:**
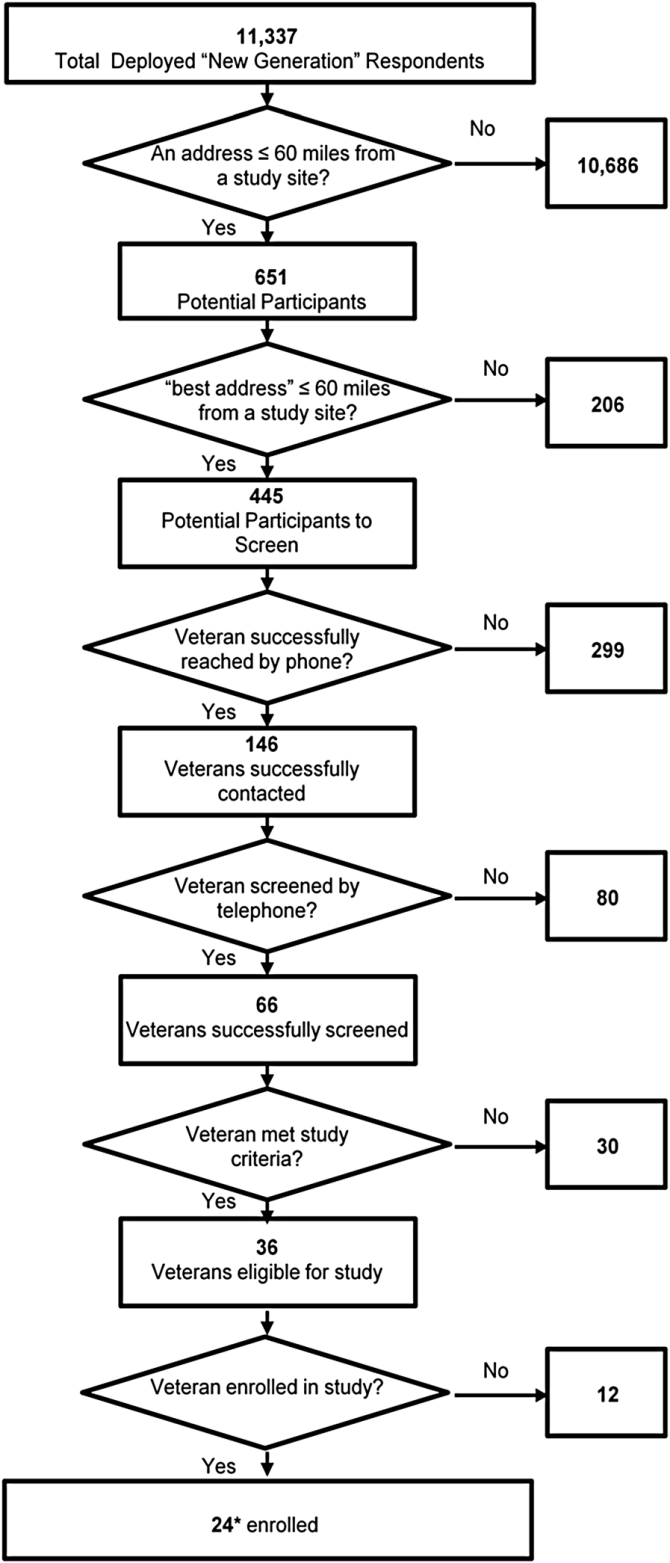
**Flowchart of each stage of the screening process.** *One participant withdrew consent. Final enrollment n = 23. Note: the 11,337 total deployed “New Generation” sample was derived from a larger sample of 13,162 respondents after removing individuals who did not meet the age criteria (18- 50 years).

A total of 13,162 deployed Veterans responded to the New Generation survey. Of these respondents, 11,337 met the age criteria for the MIND pilot study and were considered for inclusion in the current study (see screening flowchart, Figure 1). Further, selecting only those who had a high probability of living within 60 miles from one of the study sites yielded a total of 445 potential participants. This sample (n = 445) was compared to the full sample of deployed veterans from the New Generation Study (n = 13,162). Despite the exclusion of veterans >50 years old, the two samples were not statistically different in age or gender (p’s > .05). A chi-squared test revealed a significant difference between the two samples in putative diagnostic groups (χ2 (3) = 47.8, p < .001). Examination of residuals showed the MIND pilot sample had significantly fewer individuals with a positive screening result for TBI (11% vs. 16%) or PTSD (9% vs. 16%) relative to the New Generation Sample (p’s < .05). Furthermore, the MIND pilot sample had significantly more individuals with a positive screening result for combined TBI and PTSD relative to the New Generation sample (15% vs. 7%) (p < .05). The proportions of individuals with neither TBI nor PTSD were statistically similar across the MIND pilot and New Generation studies (65% vs. 61%, respectively).

Table 2 shows that the 445 individuals were unevenly distributed across the three study sites with the majority of veterans located near the Washington, D.C. and New Jersey sites. Screening calls successfully reached 146 individuals representing 33% of the target sample who were asked to participate in the screening process (Table 2). There were no statistical differences between the contacted (n = 146) and not contacted (n = 299) groups based on demographic variables (age, gender) or putative diagnostic category taken from the New Generation Study (all p’s > .05). As shown in Table 1, many of the individuals who were initially contacted were not screened (n = 80, 55%) due to either a stated lack of interest in the study or an inability to subsequently contact them despite their stated interest.

**Table 2:** Frequency of study sample who were living ≤ 60 miles from the three study sites

**Study site**	**n**	**Veterans successfully contacted**	
		**n**	**% sample**
CA	67	28	41.8
DC	189	72	38.1
NJ	189	46	24.3
Sum	445	146	
Average	148.3	47.7	34.7

“Veterans successfully contacted” refers to the frequency of people who talked directly to a screener.

To investigate possible bias in screening participation, we compared the individuals who were screened (n = 66) to the individuals who were contacted but not screened (n = 80). Five factors were considered: putative diagnostic category taken from the New Generation Study (probable TBI, probable PTSD, probable PTSD and TBI, probable neither PTSD nor TBI), age, gender, recruitment site, and distance between home address and nearest study site. Chi-squared analysis revealed that fewer individuals with a positive screening result for TBI in the absence of PTSD from the New Generation Study were screened than would be expected by chance alone (see Table 1). It is interesting to note that if the TBI group included all those with probable TBI alone or probable PTSD and TBI (n = 19), then there was no statistically significant difference in completing screening based on TBI status (χ^2^ (2) = 0.16, p > .05). None of the other four factors were significantly related to whether an individual was screened (see Table 3).

**Table 3:** Demographic comparisons between the group who was screened and the group who was not screened

**Variable**	**Screened (n = 66)**	**Not screened (n = 80)**	**Contrast**	**df**	**p**
Recruitment site					
CA (n)	12	16	χ^2^ = 1.42	2	>.05
NJ (n)	18	28			
DC (n)	36	36			
% female	17.5%	22.5%	χ^2^ = 1.42	1	>.05
Age (M ± SD)	40.2 (± 7.7)	39.8 (± 8.0)	t = -.26	144	>.05
Miles from study site (M ± SD)	27.6 (± 15.8)	25.8 (± 15.8)	t = -.69	144	>.05
Driving miles from study site (M ± SD)	38.0 (± 22.4)	34.8 (± 20.9)	t = -.90	144	>.05

Of the 66 individuals who were screened, 36 were determined to be eligible for the study. The factors associated with ineligibility generally involved exclusionary criteria including non-deployment TBI, poor cognition, OEF/OIF deployment < 30 days, and age > 50 years (see Table 4).

**Table 4:** Factors conferring study ineligibility at screening

**Reason for ineligibility following screening**	**n**	**%**
Non-deployment TBI	9	30%
Poor cognition	7	23.3%
Deployed to OEF/OIF for < 30 days	5	16.7%
> 50 years old	3	10%
Revoked interest post screening	2	6.7%
Sub-threshold PTSD	2	6.7%
Knows MIND team member	1	3.3%
Pregnant	1	3.3%
Total	30	

Twenty-four of the 36 individuals were enrolled into the study (Figure 1). Table 5 lists the reasons eligible individuals were not enrolled. Significant gender differences were found between the enrolled vs. not enrolled groups whereby fewer females than males were enrolled (Fisher’ exact test, p < .009) (group enrolled = 9% female vs. group not enrolled = 50% female).

**Table 5:** Enrollment status of screened individuals as a function of group membership in the New Generation Study

		**New generation group**				**Total**
		**TBI**	**PTSD**	**Both**	**Neither**	
Eligible (n) (%)		1 (2.8%)	5 (13.9%)	9 (25.0%)	21 (58.3%)	36 (100%)
Enrolled (n)		1	3	7	13	24
Reason not enrolled (n)	Lost contact	0	2	0	4	6
	Failed to follow up	0	0	1	1	2
	Failed to keep appointment	0	0	1	1	2
	Schedule conflict	0	0	0	2	2

Note that New Generation diagnostic categories were determined by answers to self-report screening items and not clinical diagnoses.

Of note, only two of the participants were enrolled had proactively contacted the study site after receiving the Advance Letter. In addition, the safety plan and response algorithm were not needed on any call at any of the three study sites.

## Discussion

Twenty-four veterans were recruited into a multi-site, multi-modal, observational pilot study of TBI and PTSD from a total pool of 11,337 and a likely pool of 445 participants. Between-sample comparisons revealed the two samples differed in putative diagnostic category taken from the New Generation Study. However, these statistical differences were relatively modest (the largest difference between the current study and New Generation Study was in the combined TBI and PTSD groups; 15% vs. 7%, respectively), and were driven by the large numbers involved. Challenges associated with locating, contacting, screening, and enrolling emerged. The selection of the population-based sample was based upon having previously participated in the “New Generation Study” and having been deployed to OEF/OIF conflicts. Of the 445 potential participants who were identified from records as living proximally (≤ 60 miles) to one of the three study sites, we successfully contacted 146 (contact rate = 33%). Of these, 24 were enrolled into the study, representing a recruitment yield (enrolled/total identified) of 5.4%. Factors restricting enrollment were potentially related to issues inherent to clinical research including difficulties achieving or maintaining contact with potential participants (possibly due to outdated contact information), challenges in recruiting individuals with TBI alone, the requirement to travel to the study site, and strict exclusionary criteria. Enrollment may also have been limited by the method of telephoning prospective participants who had not specifically asked to be contacted by a screener.

The recruitment yield (enrolled/total identified) of 5.4% achieved in this study is consistent with other published studies involving large civilian multi-site studies which have reported yields ranging from 0.5% [23], 1.1% [24], 2.6% [25], 5.5% [26], to 13% [27]. However, none of the aforementioned studies involved veterans or used population-based sampling methods and therefore generalization to the current study is not straightforward. Recruitment yields similar to ours have been reported for veteran studies using population-based sampling. For instance one study of Gulf War veterans [6] involving 21 VA study sites reported an overall recruitment yield of 3.5%. However, another larger study involving population-based sampling of Gulf War veterans by Eisen (2005) [28] reported a total sample of 2189 veterans enrolled from a population of 4879, representing a recruitment yield of 44.9%. The better recruitment yield in the latter study relative to ours is not likely due to demographic differences between the populations, which were quite similar. Rather, the difference probably reflects divergences in resources– for example, the Eisen (2005) study provided hotel accommodation for all participants. Of additional note, the Eisen (2005) study sample had a contact rate of 85.8%, compared to the 33% rate in the current study. This higher contact rate may reflect factors such as the relative accuracy of contact information, temporal shifts in awareness and willingness to participate in research studies, changes in telecommunication use between these studies (mobile phones vs. land lines), a lack of accurate mobile phone directories, differences in the duration and impact of the conflicts involved that may influence motivation to take part in subsequent research studies (i.e., months of engagement in the Eisen study versus years in the current study), media coverage of the issues related to Gulf War illnesses and the intense scrutiny of health issues that began shortly after the 1991 Gulf War.

In the present study, one of a number of factors associated with recruitment was our ability to contact potential participants. Of the 445 individuals identified as being eligible for the study, 67% of them were not contacted for a variety of reasons. An even larger proportion of potential participants (84%) were contacted but failed to enroll, suggesting the study population was either unable or unwilling to engage in what was offered. The largest group in this category stated they were “not interested” in the study (47%, n = 37). The reasons behind their unwillingness were not recorded but may have been due to perceived low compensation rate ($200), difficulty in committing to a full day of study, no medical treatment being offered, or a combination of factors. One solution would be to increase compensation rates. A recent review [29] notes the lack of agreement on standards of participant compensation by IRBs, who are primarily obligated to minimize coercion or undue influence. Even if recruitment was improved by increasing participant compensation, it should be noted that this may have biased the study sample. Assessing whether compensation significantly influenced epidemiological sample characteristics would be hard to determine. It was also notable that only two potential participants contacted the study sites to inquire about participation without first being called. This observation demonstrates the importance of follow-up telephone contact with potential participants.

Analysis revealed that fewer TBI cases were screened than expected. However, if these individuals were grouped together with those with TBI and PTSD, this effect disappeared. The reasons for this are not clear, but may be due to the small sample size of the “pure” TBI group (n = 4) rather than their diagnostic category. Individual characteristics such as age, gender, and distance from study site were not associated with a veteran being screened. Enhancing numbers of patients in specific subgroups who are less likely to engage would not undermine the value of population-based sampling, which should have as a primary concern recruitment of sufficient numbers of participants to enable group comparisons [7]. A recent review [30] considered the nature of barriers to participation in mental health research and identified many factors including transportation difficulties, distrust and suspicion of researchers, and the stigma attached to mental illness. The authors concluded that strategies to overcome these barriers included assistance with travel, avoiding the use of stigmatizing language in study materials and an emphasis on education about the disorder under investigation. Considering the recent increase in prevalence of PTSD and TBI in veterans [31,32], more studies that systematically investigate recruitment strategies are needed in veteran populations with these disorders.

The observation that major factors in recruitment were the ability to contact participants and enroll them suggests a need for improved methods of communication with potential participants. Researchers are encouraged to consider novel approaches to establish contact, develop rapport, and secure a commitment to participate. Such approaches include communication technologies such as telehealth, internet, videoconferencing, email, and text messaging [33]. In this regard, it should also be noted that only 3.4% of potential participants lived within a 60-mile radius of one of the three study sites. Recruiting the remaining 96.6% of potential participants into the study would take considerable resources to bring them to the study center and to accommodate them. If these resources were not available, alternative study designs could be considered including the use of communication technologies instead of site visits. Communication technologies therefore offer an important alternative to an on-site evaluation, especially for participants who live remotely from study sites.

The current analysis represents a detailed report of recruitment metrics for a population-based sampling strategy. The VHA, Centers for Disease Control, National Center for Health Statistics, and Department of Defense all have several ongoing studies using population-based sampling methods but few published data exist describing the challenges of recruiting participants and the characteristics associated with contact, screening, and enrollment. One exception is the Millennium Cohort Study, which is a 22-year longitudinal study launched in 2001 to determine how military service affects long-term health [34]. The study uses several innovative procedures to contact, communicate with [35] and retain [36] large numbers of veteran participants. Our report takes advantage of clear criteria for each stage of the enrollment process from a well-described, representative sample allowing for more detailed assessment of bias in responses. One significant limitation of the current study is its small sample size. Despite this, we believe that our main findings can better inform the conduct of future clinical investigations using population-based sampling.

## Conclusion

In conclusion, the screening methods, recruitment procedures, and enrollment numbers from this study using population-based sampling can help guide recruitment for future studies. Clearly, to achieve adequate sample sizes, a large pool of potential participants must be available, and adequate resources must be devoted to enhance participation. Exploration of solutions that overcome barriers to participation, including travel time, lack of interest, and failure to keep a study appointment, is warranted. Some of these issues may be universally relevant, while others (some unknown) may arise in particular studies.

## Abbreviations

IRB: Institutional Review Board; MIND: Markers for the Identification, Norming and Differentiation of TBI and PTSD study; OEF: Operation Enduring Freedom; OIF: Operation Iraqi Freedom; PTSD: Post Traumatic Stress Disorder; TBI: Traumatic Brain Injury; VA: Veterans Affairs; VHA: Veterans Health Administration; WRIISC: War Related Illness and Injury Study Center.

## Competing interests

The authors declare that they have no competing interests.

## Authors’ contributions

PJB supervised the data collection, performed statistical analysis and drafted the manuscript. JYK carried out data collection and analysis and helped draft the manuscript, DAH supervised data collection, participated in study conception and design and helped draft the manuscript, AS participated in study design, provided patient data and helped draft the manuscript, LAR participated in study design, and collected data, SMR participated in study design, and collected data, JAJ participated in study design, and collected data, JB participated in study design, collected data, and administered IRB permissions, LI collected data and helped draft the manuscript, MN participated in study design, and collected data, MRB participated in study conception and design and helped draft the manuscript, MJR participated in study conception and design, helped draft the manuscript and supervised data collection, JWA participated in study conception and design, helped draft the manuscript and supervised data collection, JCC participated in study conception and design, helped draft the manuscript and supervised data collection. All authors read and approved the final manuscript.

## Authors’ information

**The MIND Study Group:** Julie C. Chapman, Aaron Schneiderman, Matthew J. Reinhard, J. Wesson Ashford, Drew A. Helmer, Gudrun Lange, Helena K. Chandler, Maheen M. Adamson, Peter J. Bayley, Jamie M. Zeitzer, Jerome Yesavage, Marc R. Blackman.

## Pre-publication history

The pre-publication history for this paper can be accessed here:

http://www.biomedcentral.com/1471-2288/14/48/prepub

## Supplementary Material

Additional file 1 **Inclusion and exclusion criteria.** Inclusion and exclusion criteria used in the MIND pilot study. Click here for file
