# Supplementary material for: Challenges to be overcome using population-based sampling methods to recruit veterans for a study of post-traumatic stress disorder and traumatic brain injury
Source: BMC Med Res Methodol. 2014 Apr 8;14:48. doi: 10.1186/1471-2288-14-48 (PMC4101880; doi:10.1186/1471-2288-14-48)
Supplement: Additional file 1 — Inclusion and exclusion criteria. Inclusion and exclusion criteria used in the MIND pilot study. [file 1471-2288-14-48-S1.docx]

**Additional File**

*Inclusion criteria*

Participated in the “New Generation Study”

Patient deployed for ≥30 days in OEF/OIF conflict

Age 18 - 50 years

*Exclusion criteria*

Weight >375lbs

Education <10 years

Patient is a woman who is pregnant

Patient believes it would be difficult not to drink alcohol or use drugs during visit to study site

Patient is currently suicidal or actively thinking about harming themselves

Open head wound sustained during deployment

Head injury during deployment < one year ago

Patient lost consciousness >30 mins due to head injury

Symptoms of traumatic brain injury (dazed, amnesia, sensitivity to bright light, irritability, headache, sleep problems, trouble concentrating, hearing problems) sustained outside deployment did not resolve in one week

Inability to undergo MRI (e.g., metal objects in body, claustrophobia, etc.)

Visual problems that interfere with ability to read or write

Problems with hands/arms that prevent use of pencil, computer mouse or keyboard

Patient has received a professional diagnosis of severe neurological or psychiatric illness (e.g., Parkinson’s disease, Bipolar disorder, Narcolepsy, etc.)
